# Concurrent fNIRS and EEG for Brain Function Investigation: A Systematic, Methodology-Focused Review

**DOI:** 10.3390/s22155865

**Published:** 2022-08-05

**Authors:** Rihui Li, Dalin Yang, Feng Fang, Keum-Shik Hong, Allan L. Reiss, Yingchun Zhang

**Affiliations:** 1 Center for Interdisciplinary Brain Sciences Research, Department of Psychiatry and Behavioral Sciences, Stanford University School of Medicine, Stanford, CA 94305, USA; 2 Department of Biomedical Engineering, University of Houston, Houston, TX 77004, USA; 3 School of Mechanical Engineering, Pusan National University, Pusan 43241, Korea; 4 Mallinckrodt Institute of Radiology, Washington University School of Medicine in St. Louis, 4515 McKinley Avenue, St. Louis, MO 63110, USA

**Keywords:** EEG, functional NIRS, multimodal neuroimaging, concurrent recording, integrated analysis

## Abstract

Electroencephalography (EEG) and functional near-infrared spectroscopy (fNIRS) stand as state-of-the-art techniques for non-invasive functional neuroimaging. On a unimodal basis, EEG has poor spatial resolution while presenting high temporal resolution. In contrast, fNIRS offers better spatial resolution, though it is constrained by its poor temporal resolution. One important merit shared by the EEG and fNIRS is that both modalities have favorable portability and could be integrated into a compatible experimental setup, providing a compelling ground for the development of a multimodal fNIRS–EEG integration analysis approach. Despite a growing number of studies using concurrent fNIRS-EEG designs reported in recent years, the methodological reference of past studies remains unclear. To fill this knowledge gap, this review critically summarizes the status of analysis methods currently used in concurrent fNIRS–EEG studies, providing an up-to-date overview and guideline for future projects to conduct concurrent fNIRS–EEG studies. A literature search was conducted using PubMed and Web of Science through 31 August 2021. After screening and qualification assessment, 92 studies involving concurrent fNIRS–EEG data recordings and analyses were included in the final methodological review. Specifically, three methodological categories of concurrent fNIRS–EEG data analyses, including EEG-informed fNIRS analyses, fNIRS-informed EEG analyses, and parallel fNIRS–EEG analyses, were identified and explained with detailed description. Finally, we highlighted current challenges and potential directions in concurrent fNIRS–EEG data analyses in future research.

## 1. Introduction

The human brain comprises billions of neurons [1]. Each of these forms a number of synapses, establishing a complicated network with quadrillions of connections and thus enabling our brains to function in an adaptive manner [2]. Although our understanding of neurons on a microscopic scale has progressed in recent decades, little is known about how these huge numbers of neurons (and synapses) work collectively to generate macroscopic brain signals and human behaviors. It is believed that human brain functions and associated behaviors are carried out by complex neural activations and networks. These internal activities generally elevate electrical activity (direct effects) accompanied by a hemodynamic and metabolic response (indirect effects), which serve as the basic sources for all noninvasive neuroimaging techniques. Depending on the sources of the signals, these brain imaging techniques can be roughly divided into two categories. The first category refers to imaging techniques that directly capture the neural electrical activities by detecting the induced electrical or magnetic fluctuations over the scalp. The most representative methods in this category are Electroencephalography (EEG) and Magnetoencephalography (MEG). The second category comprises indirect imaging approaches that rely on hemodynamic (cerebral blood flow, cerebral blood volume) and metabolic (glucose and oxygen utilization) responses induced by neural activity. Commonly available techniques in this category include functional near-infrared spectroscopy (fNIRS), functional magnetic resonance imaging (fMRI), and positron emission tomography (PET). In this perspective, EEG and fNIRS have been gaining popularity in the research community and clinical practice due to their distinct natures, particularly their noninvasiveness, mobility, and flexibility.

### 1.1. The Fundamental Basis of fNIRS

Functional Near-infrared Spectroscopy (fNIRS), first reported by Jobsis in 1977 [3], is an optical imaging technique for non-invasive investigation of hemodynamic responses in the brain. fNIRS usually utilizes lights with distinct wavelengths (between 600 and 1000 nm) that can penetrate the scalp and reach the cortical surface to measure the concentration changes of oxygenated hemoglobin (HbO) and deoxygenated hemoglobin (HbR) that are coupled with the metabolic activity of neurons in the outer layers of the cortex. This technique is particularly useful for studying the functional activation within the brain due to the inherent relationship between neural activity and hemodynamic responses in the brain [4]. Specifically, fNIRS measures the regional changes of HbO and HbR concentration, which can serve as an indicator of hemodynamic changes associated with neural activity in the brain.

**Figure 1:**
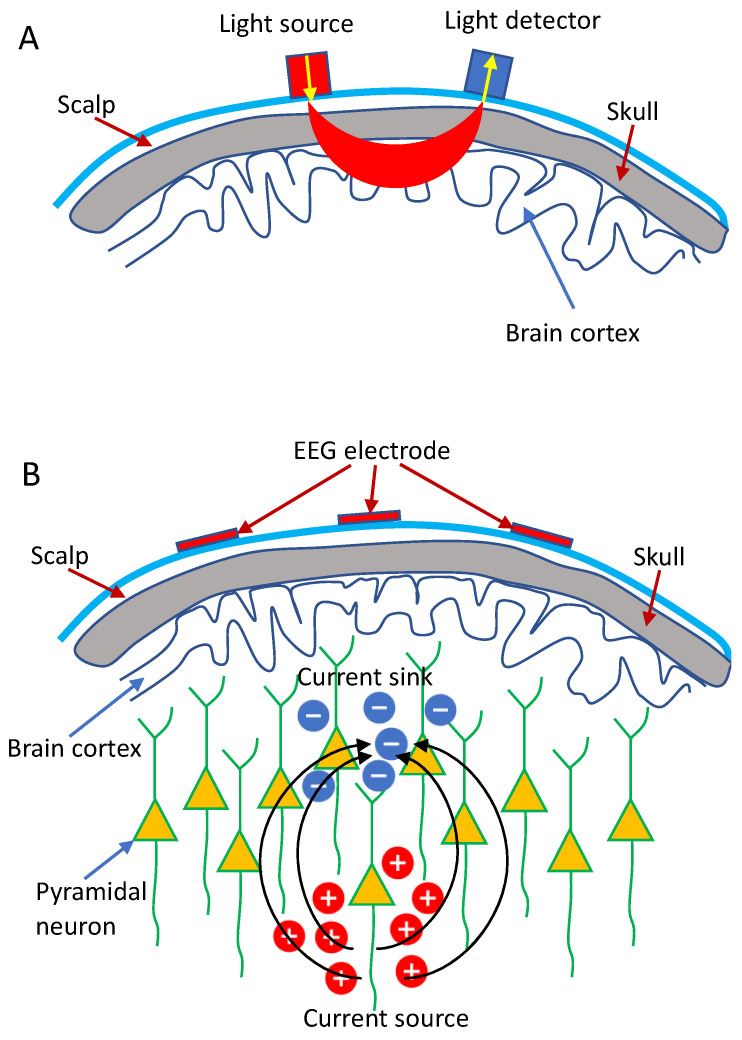
Schematic demonstration: (**A**) fNIRS and (**B**) EEG measurement.

Currently, the continuous wave NIRS (CW-NIRS) is extensively used in the research and clinical settings due to its low cost and simplicity. The measurement of the hemoglobin concentration (HbO and HbR) in CW-NIRS primarily relies on the physical basis that chromophores inside the brain, especially the HbO and HbR, have specific and sensitive absorption characteristics in the near-infrared range (between 600 and 1000 nm). Lights at different wavelengths can then be injected into the brain via the sources (illuminators) placed on the scalp, and the attenuated lights are detected by the optical detectors placed near the illuminators (Figure 1A), from which the concentration changes of HbO and HbR can be computed based on the Modified Beer-Lambert Law [5]. Specifically, CW-NIRS systems typically utilize laser/LED sources to shine two distinct wavelengths into the brain at a constant intensity and use detectors to measure the intensity of diffusely reflected light continuously.

### 1.2. The Fundamental Basis of EEG

Electroencephalography (EEG), first described by Hans Berger in 1929 [6], is thought to result primarily from the synchronization of post-synaptic potentials at cortical pyramidal neurons [7]. The recorded EEG signal does not represent single neuron depolarization inside the brain. Instead, it is assumed that tens of thousands of synchronized pyramidal neurons within the cortex are firing when the brain is activated, wherein dendritic trunks of the neurons are coherently orientated, parallel with each other and perpendicular to the cortical surface so as to induce sufficient summation and propagation of electrical signals to the scalp (Figure 1B) [8].

Typically, EEG signals are measured through EEG electrodes (including a reference electrode and a ground electrode) placed over a subject’s scalp. Voltage differences between the electrodes and the reference electrode are then measured and amplified (Figure 1B). The recorded EEG signals, which represent the large-scale neural oscillatory activity, can be divided into various rhythms depending on characteristic frequency bands, including theta (4–7 Hz), alpha (8–14 Hz), beta (15–25 Hz), and gamma (>25 Hz) [9]. These brain rhythms contain information associated with the ongoing neuronal processing in specific brain areas, which allows EEG to be used as a non-invasive method for the characterization of cortical reorganization, induced by various brain disorders, particularity in the diagnosis of epilepsy and stroke [9,10,11,12], and the assessment of brain state alterations [13,14,15].

### 1.3. Integration of EEG and fNIRS: Rationale and Advantages

The functional activity of the cerebral cortex can be investigated using various imaging techniques including EEG, fNIRS, fMRI, and their combinations [16,17,18,19]. Each of these techniques has its own advantages and disadvantages. However, single-modality imaging techniques can only capture limited information associated with neural activity due to their technical limitations and the inherent complexity of neural processing within the brain. For example, compared to fMRI, fNIRS features higher temporal resolution (<1 s), good portability, lower cost, good resistance to motion artifacts, and applicability to various measurement scenarios including clinical settings as well as the natural environment [5]. More importantly, fNIRS measurements have been proven to be similar to the blood oxygen level dependent (BOLD) response obtained by fMRI [20]. However, there are also several limitations of fNIRS techniques: the limited penetration depth, low signal-to-noise ratio, and low temporal resolution compared to EEG. EEG possess several advantages over fMRI for exploring dynamic brain activity: it is portable, inexpensive, and features a remarkably high temporal resolution (millisecond) compared to fNIRS and fMRI [21], though EEG is highly vulnerable to motion artifacts that would inhibit the EEG measurement in a natural settings [22].

To comprehensively explore the functional activity of the brain, multimodal approaches are needed. Integrated EEG–fNIRS approaches offer numerous benefits over single-modality methods by exploiting their individual strengths; EEG provides favorable temporal resolution while fNIRS offers better spatial resolution and is robust to noise [23,24]. Additionally, EEG and fNIRS signals are associated with the neuronal electrical activity and metabolic response, respectively, providing a built-in validation for identified activity. Measurements obtained from each of these two modalities thereby provide complementary information related to functional activity of the brain.

**Figure 2:**
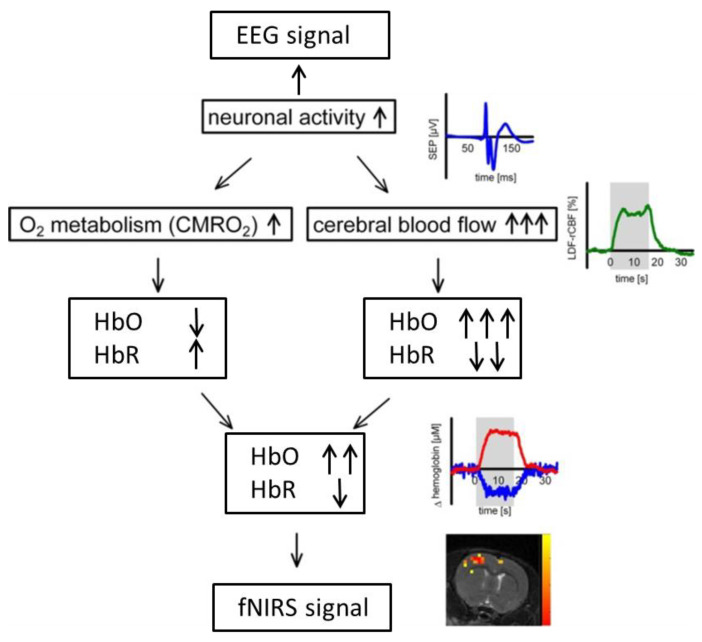
Demonstration of neurovascular coupling.

In addition to their complementary technical properties, the rationale behind the combination of EEG and fNIRS relies on a physiological phenomenon called neurovascular coupling within the brain [25]. Neural activity is inherently accompanied with the fluctuation of cerebral blood flow (CBF) that carries vital oxygen and nutrients to neurons. Specifically, when neurons are activated within a specific brain region, blood will flow to that brain region to meet the increased demand of glucose and oxygen, resulting in fluctuations of hemoglobin concentration (HbO and HbR) that can be detected by functional imaging techniques such as fNIRS and fMRI (Figure 2). The so-called neurovascular coupling forms the theoretical basis for integrated fNIRS–EEG imaging of brain activity. It has been shown in recent studies that impairment of neurovascular coupling could serve as a sign for several neurological diseases such as Alzheimer’s disease and stroke [25,26,27], which might provide a new prospective for evaluation and diagnosis of neurological diseases as well as increase our understanding of mechanisms underlying neurovascular coupling.

### 1.4. Motivation of the Present Review

The fact that integration of fNIRS and EEG provides complementary information about electrical and metabolic-hemodynamic activity of the brain activity has led to increasing investigations of the benefits of integrated EEG and fNIRS [27,28,29]. In the last decade, numerous studies utilizing integrated fNIRS–EEG systems have been reported on both nonclinical and clinical topics [30]. Data analysis of concurrent fNIRS–EEG recordings is a fundamental but essential step for fNIRS–EEG research studies. This step usually consists of several key processes, including raw data processing, feature extraction, and integrated/fused analysis of these two modalities. Although several recent reviews have been published to summarize the latest progress on applications of concurrent fNIRS–EEG recordings, such as brain–computer interface, development of wearable fNIRS–EEG devices, and neuromodulation, there is no comprehensive summary yet regarding the general analysis pipeline of simultaneously recorded fNIRS and EEG signals. To fill this knowledge gap, this review aims to systematically summarize the status of analyses methods used in concurrent fNIRS–EEG studies involving healthy individuals as well as patient populations. Specifically, we focus on multiple levels of integrated analyses of concurrent fNIRS–EEG recordings by critically evaluating the data processing methods, extracted features, and forms of integration of these two modalities. The present review differs from previous reviews in that this is the first systematic, methodology-focused review to describe which approaches were used in previous concurrent fNIRS–EEG studies and how these approaches were used, thus providing an up-to-date overview and technical guideline for future projects to conduct concurrent fNIRS-EEG studies.

This review is organized as follows: Section 1 is dedicated to the description of the origins, the main characteristics of fNIRS and EEG, and the rationale of combining fNIRS and EEG for multimodal brain imaging. Section 2 describes the strategy of our literature review and the criteria of identification and classification of published articles. Section 3 starts with a brief summary of the preprocessing of raw fNIRS and EEG data and then elaborates three main categories of analysis approaches in concurrent fNIRS–EEG studies. Finally, Section 4 is devoted to underlining the limitations, challenges, and future direction of data analysis of integrated fNIRS–EEG techniques.

## 2. Methodology

**Figure 3:**
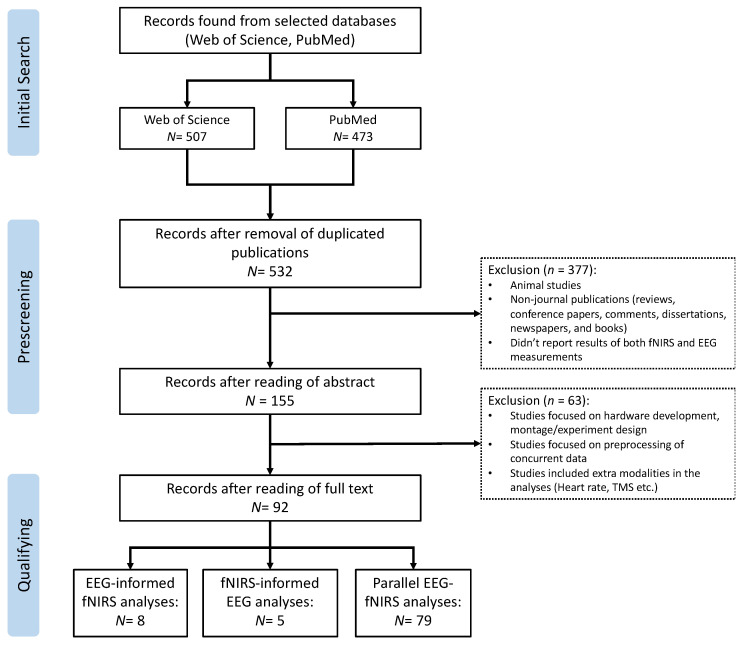
PRISMA flow diagram for the literature review and article selection.

This review was conducted following the Preferred Reporting Items for Systematic Reviews and Meta-Analyses (PRISMA) protocol [31]. As shown in Figure 3, the flow diagram of PRISMA mainly includes three steps: (1) initial search: search related studies based on the defined keywords in selected databases; (2) prescreening: remove duplicated articles and select articles based on designed criteria; (3) qualifying: read through the full text of the selected articles to make sure they meet the eligibility and inclusion criteria.

### 2.1. Search Strategy

The search for relevant peer-reviewed articles describing the use of a concurrent fNIRS–EEG design was conducted on PubMed and Web of Science as literature sources. The following keyword combinations were used in the literature search: (“fNIRS” OR “NIRS” OR “functional near-infrared spectroscopy” OR “near-infrared spectroscopy”) AND (“EEG” OR “electroencephalography”) AND (“Brain”). Only articles that were published in English through 31 August 2021 were included.

### 2.2. Prescreening and Qualifying Criteria

The prescreening criteria were based on the reading of titles and abstracts. First, duplicated articles under different titles were removed. Then, publications were excluded if they (1) were not in line with the topic, i.e., animal studies; (2) were non-journal publications, such as reviews, conference papers, comments, dissertations, newspapers, and books; and (3) did not report analysis results of both fNIRS and EEG measurements.

We then performed further screening and qualifying by reading through the full text of the articles. In this process, publications were excluded if they (1) focused on montage design, experimental design, or hardware development of concurrent fNIRS–EEG systems; (2) focused on preprocessing of fNIRS and/or EEG data; or (3) included extra modalities in the analyses, such as heart rate, electromyography, transcranial magnetic stimulation, etc. Furthermore, the following inclusion criteria for the review were considered: (1) articles focusing on brain function investigation using concurrent fNIRS–EEG were included; (2) articles with details of signal processing, feature extraction, and concurrent analysis of fNIRS–EEG were included.

## 3. Results

**Figure 4:**
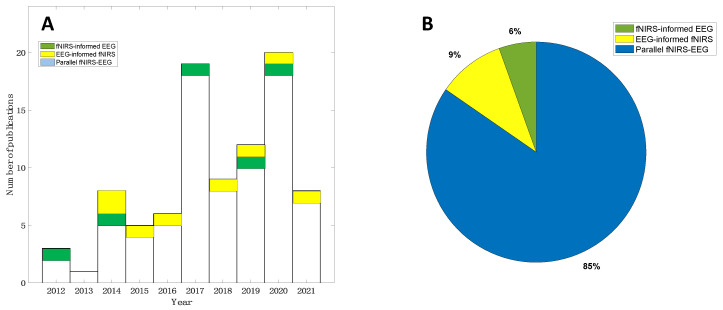
Literature summary of concurrent EEG–fNIRS studies: (**A**) Yearly publications from 2012 to 2021 and (**B**) distribution of each type of concurrent fNIRS-EEG studies.

The search strategy resulted in a total of 980 records in the initial search from the selected databases (507 from Web of Science and 473 from PubMed, Figure 3). After the prescreening and qualifying stages, we obtained a total of 92 articles available for this review, including 5 studies focusing on fNIRS-informed EEG analyses, 8 studies focusing on EEG-informed fNIRS analyses, and 79 studies focusing on the parallel analyses of fNIRS–EEG (Figure 3). Figure 4A summarizes the number of concurrent fNIRS–EEG studies each year since 2012, and Figure 4B shows the percentage of each type of integrated analysis of fNIRS–EEG.

### 3.1. Preprocessing of fNIRS and EEG Signal

Signal preprocessing is an essential step for any post-processing of integrated analysis of concurrent fNIRS–EEG data. Since the present review specifically focused on the integrated analysis of concurrent fNIRS–EEG data, here we only outline a general pipeline for the basic preprocessing of each modality.

#### 3.1.1. Basic Preprocessing of fNIRS Signal

**Figure 5:**
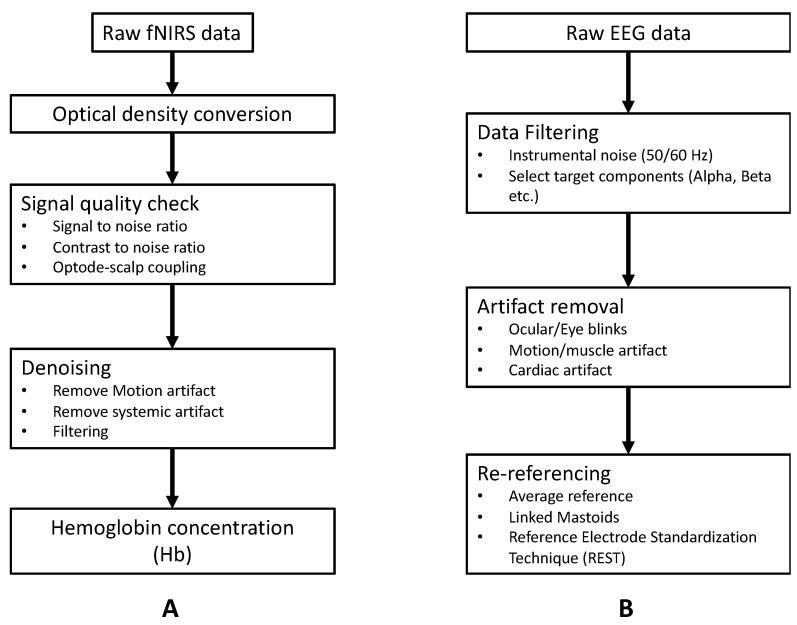
Basic preprocessing pipeline: (**A**) fNIRS raw data and (**B**) EEG raw data.

Basic preprocessing of fNIRS data is shown in Figure 5A. One particularly essential step in the preprocessing of fNIRS data is signal quality check and artifact correction. The quality of the fNIRS signal could be affected by several confounding noise sources, such as instrument noise (e.g., due to light source instability, electronic noise) [32], physiological interference (e.g., respiration, heartbeat) [33,34], or motion artifacts [35,36]. Instrument noise and physiological interference are mostly located within a constant frequency range. For instance, the instrument-degradation-induced noise is around 3~5 Hz, and respiration and heartbeat lie in 1~1.5 Hz and 0.2~0.5 Hz, respectively [5]. Thus, these noises can be easily removed by applying the band-pass filter/low pass filter. Motion artifact in the form of spikes or baseline shifts is a typical category of noise in raw fNIRS signal, especially in data collected from child populations or during experimental tasks that include motion (e.g., walking or speaking) [36,37]. Multiple algorithms have been developed to identify and correct motion artifacts in raw fNIRS signals, such as spline interpolation [38], wavelet-based methods [39,40], or principal component analysis [41]. We refer the readers to recently published articles for a more detailed overview of the preprocessing of fNIRS signal [42,43].

#### 3.1.2. Basic Preprocessing of EEG Signal

We have outlined the basic preprocessing of EEG data in Figure 5B. Similar to fNIRS, EEG recordings are often contaminated by different artifacts that come from internal and external sources. Internal artifacts include physiological activities of the subject (e.g., ECG, muscle, and ocular artifacts) and movement [44,45]. External artifacts mainly include environmental/instrumental interference (50 Hz/60 Hz), electrode pop-up and cable movement. Elimination of internal artifacts relies on extra measurements (e.g., electrooculogram/electrocardiogram/accelerometer) or signal decomposition algorithms (e.g., ICA/PCA) [46,47]. External artifacts may be removed either by simple filters, signal decomposition algorithms (e.g., ICA), or artifactual segment rejection [48]. We refer the readers to [49,50] for a more detailed overview of the preprocessing of EEG signals.

### 3.2. EEG-Informed fNIRS Analyses

Neurovascular coupling demonstrates that regional neural activity is typically accompanied by the generation of electrical activity and the resulted metabolic variation, which is the fundamental principle of EEG and fNIRS measurements. Simultaneous fNIRS–EEG recording is therefore highly suited for neurovascular coupling investigation through various analysis approaches.

Among all the concurrent fNIRS–EEG studies, using EEG-derived characteristics to enhance fNIRS analyses, which is usually referred as EEG-informed fNIRS analyses, provides a particularly new and straightforward solution for investigating neurovascular coupling. Table 1 summarizes all studies that performed EEG-informed fNIRS analyses.

**Figure 6:**
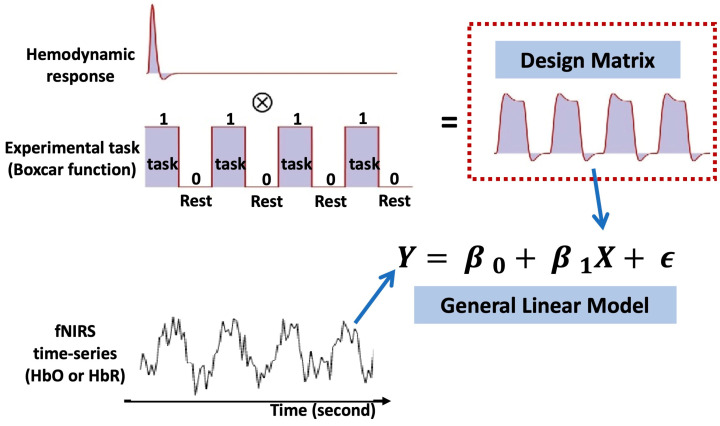
Basic principle of general linear model (GLM) in fNIRS analysis.

In typical fNIRS analyses (Figure 6), the fNIRS signal is commonly regressed via a general linear model (GLM) constructed by convolving the canonical hemodynamic response function (HRF) with a boxcar or impulse function representing the consistent temporal profile of the experimental paradigm to identify cortical regions activated by specific stimuli [59]. Briefly, for measured fNIRS signal Y in a channel, the GLM model is given by

(1) Y = Xβ+ε

where X is the design matrix, β is the regression coefficients to be estimated, and ε is the error term. In the case of a block design experiment, X is commonly given by a convolution matrix of a chosen hemodynamic response function (HRF) and boxcar functions describing the latency and duration of the stimulus. Note that the HRF may use various type of shapes, such as canonical HRF, gamma-HRF, or Gaussian-HRF [35]. Columns of X are the regressors that represent conditions or tasks in the experiment, and additional nuisance terms or auxiliary measurements that usually account for the systemic physiology or motion artifacts.

The estimated regression coefficient β and the error ε can be tested via a *t*-test to identify the channels that represent a significant contrast between different tasks. The *t*-test is calculated by

(2) $$t=\frac{c^{T}\times \beta }{\sqrt{c^{T}cov\left(\beta \right)c}\;},$$

where  cov(β) is the covariance matrix of  β and c is the contrast vector, which determines the contrast between specific conditions.

The main limitation in common standalone fNIRS analysis is that neuronal response to repeated trials or stimuli is time-varying across the experiment in a realistic setting, which may be inconsistent with the boxcar function typically used in the construction of a GLM analysis design matrix. With this in mind, the core idea of EEG-informed fNIRS analysis is to replace or adjust the boxcar function in the fNIRS GLM analysis with temporal- or frequency-specific regressors of interest derived from EEG signals [57]. Based on the linear hypothesis of neurovascular coupling, the characteristics of the neural activity extracted in EEG may offer better estimation of the fNIRS response after convoluting the HRF, thus increasing the efficiency of identifying the related active region induced by experimental tasks.

**Figure 7:**
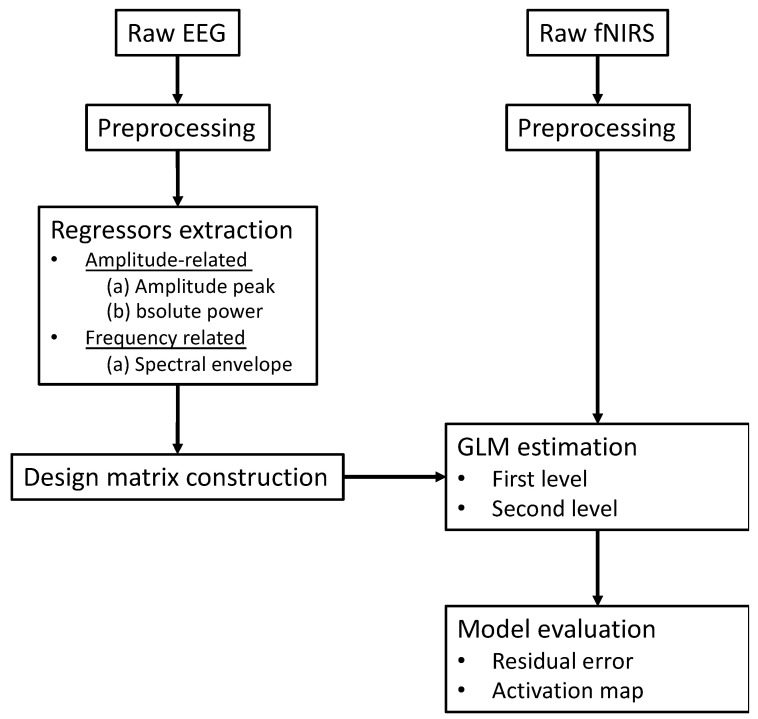
The conventional schematic of EEG-informed fNIRS GLM analysis framework.

Figure 7 summarizes a generalized analysis framework of EEG-informed fNIRS analysis. The selection of time-varying EEG features plays a crucial role in the construction of a fNIRS GLM analysis design matrix. Among all EEG-informed fNIRS analysis studies, amplitude information derived from EEG signals has been used as effective regressors of interest for improving the estimation of the active fNIRS response associated with different stimuli [56,57]. Li et al. collected concurrent EEG and fNIRS data from healthy participants during a repeated motor execution task and extracted the peak value and latency of the EEG signal within each trial to construct a series of frequency-specific design matrices [57]. Their results showed that amplitudes of frequency-specific EEG components, especially the alpha and beta band, could better capture the time-varying neural activity at single trial level and thus enhance the performance of fNIRS GLM analysis when compared with the classic boxcar function-based fNIRS method [57]. The potential value of EEG-informed fNIRS analysis in clinical applications was also explored, in particularly on the topic of epileptic activity, given the suitability of this technique for the localization of brain sites associated with epileptic discharges. A series of representative studies was performed by Pouliot and his colleagues, where the onsets and amplitudes of epileptic spikes were identified by EEG temporal traces and convolved with the HRF for fNIRS GLM estimation [51,52,54,60]. These studies demonstrated that an EEG-informed fNIRS approach revealed higher sensitivity and specificity than the classic GLM method in the detection of epileptic events such as seizures or interictal epileptiform discharges (IEDs). Their work provides evidence that EEG-informed fNIRS analysis could be a sensitive technique for monitoring epileptic activity.

In addition to the amplitude-specific information, frequency-related features were derived from EEG signals and used as regressors of interest for fNIRS GLM analysis. Talukdar et al. used gamma transfer functions to map EEG spectral envelopes that reflect time-varying power variations in neural rhythms to hemodynamics measured during median nerve stimulation [53]. The approach was evaluated through simulated EEG–fNIRS data and experimental EEG–NIRS data measured from three human subjects. Results indicated that fNIRS hemodynamics can be predicted by EEG spectral envelopes convoluted with multiple sets of gamma transfer functions, providing a new perspective for the modeling of neurovascular coupling.

### 3.3. FNIRS-Informed EEG Analyses

Studies using fNIRS to enhance the processing of EEG signals typically rely on the relatively robust spatial information of fNIRS compared to EEG. Within this context, fNIRS-informed EEG analyses, as summarized in Table 2, include two main levels of applications: fNIRS-informed EEG source localization and fNIRS-informed EEG channel selection. The former applies task-evoked information of fNIRS to enhance the mathematical estimation of active EEG source activity related to specific tasks [27], while the latter used fNIRS as a reliable reference for choosing the most representative task-related EEG channels for analysis [61].

#### 3.3.1. FNIRS-Informed EEG Source Imaging Analysis

Due to its high temporal resolution and portability, EEG is by far the most widely used neuroimaging technique to measure rapid neuronal electrical activity. However, one limitation of scalp EEG is the volume conduction problem; a single electrode on the scalp picks up activity from multitude sources (cortical activity, subcortical activity, external noise, etc.), which results in difficulty accurately localizing the source activity [62]. Therefore, EEG source imaging (ESI) has been developed to overcome the limitation of scalp EEG in characterizing the spatial brain activity. Typically, ESI relies on the surface EEG signals and the anatomical structure and physiological properties of the brain to estimate sources within the brain. This allows for more accurate localization of the cortical regions contributing to EEG signals measured at the scalp. A common challenge for ESI is the ill-posed “inverse problem”; the number of sources that give rise to EEG signals vastly outnumbers the available measurements, making it impossible to localize the measured scalp EEG activity to the actual current-generating source within the brain with absolute certainty [63]. Given the good spatial resolution of fNIRS, the majority of fNIRS-informed EEG studies have focused on using fNIRS-based spatial priors to enhance the estimation of EEG source activity.

**Figure 8:**
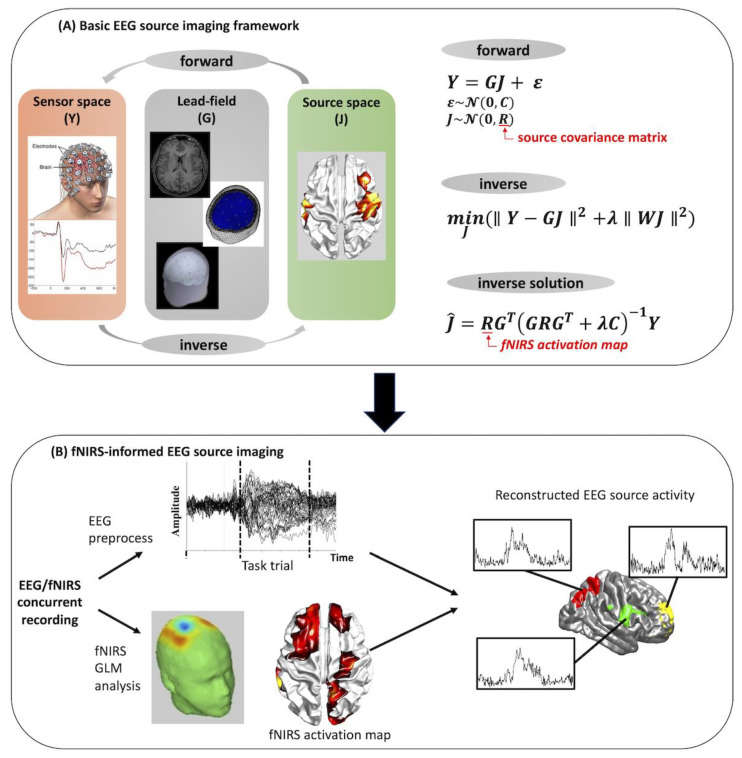
Basic concepts of EEG source imaging and traditional pipeline of fNIRS-informed EEG source imaging analysis (adapted with permission from Ref. [27]. 2019, Li et al.

In summary of these studies, a traditional pipeline of fNIRS-informed EEG source imaging is shown in Figure 8. Briefly, this pipeline begins with the forward model of the ESI (Figure 8A):

(3) Y=GJ+ε,

where $Y\;\in \;{\mathbb{R}}^{m\times d}$ is the scalp EEG signal consisting of m channels and d measurement samples, $J\in \;{\mathbb{R}}^{s\times d}$ is the unknown source activity of s dipole sources in the source space, $G\;\in \;{\mathbb{R}}^{m\times s}$ is the lead field matrix which describes the relationship between the source activity and the EEG electrodes, and ε represents the noise component in the sensor space. Using the EEG signals measured at the scalp, we can attempt to invert the forward model to determine which parts of the brain are active from their associated scalp potentials, which is the so-called inverse problem. A common solution of the inverse problem using classical minimum-norm estimate (MNE) is given as:

(4) $$\hat{J}=RG^{T}{\left(GRG^{T}+\lambda C\right)}^{-1}Y,$$

where $\hat{J}$ is the estimated source activity, R is the source covariance matrix representing the prior knowledge about the distribution of source J, C is the noise covariance matrices, and λ is the regularization parameters representing the trade-off between model accuracy and complexity, which is traditionally determined using the L-curve method [64]. The source covariance matrix R and noise covariance matrix C are usually set to identity matrices when no prior information about the source space is available. With this in mind, spatial prior information provided by fNIRS, usually represented by *t* values of significant channels after GLM analysis, can be applied directly on the source covariance matrix R, changing the weight of each source according to whether or not it is within an fNIRS-active region. This results in improvement of EEG source activity estimation (Figure 8B). Note that the inverse problem can be solved by multiple approaches, such as MNE, weighted MNE, or probabilistic Bayesian methods, resulting in different forms of source covariance matrix R [65,66].

The analysis pipeline shown in Figure 8B has been adapted in all existing fNIRS-informed EEG analysis studies to investigate brain dynamics associated with typical brain function as well as brain disorders. The first fNIRS-informed ESI study was carried out by Aihara et al., in which the authors incorporated the fNIRS-based prior information in the current source estimation using a Variational Bayesian Multimodal EncephaloGraphy (VBMEG) method [67]. Using a simulation study and a finger tapping motor task, this study demonstrated that fNIRS-informed ESI can achieve results similar to fMRI-information ESI. Following a similar idea, Morioka et al. applied fNIRS-informed ESI to decode subjects’ mental states in a spatial attention task and found that the fNIRS–EEG framework exhibited significant performance improvement over decoding methods based on EEG sensor signals alone [68]. Recently, Li et al. employed the fNIRS-informed ESI technique to explore the atypical brain dynamics associated with Alzheimer’s disease and stroke, from which brain network alterations induced by these brain disorders were characterized in a high spatiotemporal manner [27,69].

#### 3.3.2. FNIRS-Informed EEG Channel Selection for BCI Studies

FNIRS-informed EEG source imaging represents the deep fusion of fNIRS and EEG signals. In addition, one study published by Li et al. demonstrated that fNIRS-based spatial prior information can also be used to optimize processing of scalp EEG signal in BCI studies [61]. Briefly, a desirable BCI system should be portable, minimally invasive, and feature high classification accuracy and efficiency. However, the main challenge of hybrid EEG–fNIRS BCI systems is how to reduce the complexity of the system while achieving a satisfactory performance. To tackle this challenge, Li et al. proposed a fNIRS-based channel selection method to greatly reduce the number of fNIRS and EEG channels needed for BCI systems. In this fNIRS-based channel selection method, two fNIRS channels with strongest task-evoked response, as assessed by GLM analysis, were determined. Then only two EEG channels that were close to the selected fNIRS channels were selected for the performance assessment of the hybrid fNIRS–EEG BCI system. Results demonstrated that this approach could drastically minimized the burden (e.g., weight of cables, preparation time) on the user while achieving a good performance compared to BCI systems including large numbers of channels [61].

Overall, although limited studies focused on this topic were available or review, fNIRS-informed EEG source imaging analysis has potential for achieving a deep fusion of these two portable techniques. This multimodal approach holds promise for improving our understanding of the spatiotemporal dynamics of typical and atypical brain functions in various scenarios including naturalistic interaction and clinical settings.

**Table 2:** Characteristics of studies performed fNIRS-informed EEG analysis.

Authors	Tasks	Brain Regions	Features	Analysis Methods
Aihara et al., 2012 [67]	Motor (Simulation; Experiment)	fNIRS: Motor EEG: Whole	fNIRS: HbO peak EEG: Source current amplitude	EEG source imaging
Morioka et al., 2014 [68]	Mental	fNIRS: Parietal, occipital EEG: Whole	fNIRS: HbO t-statistic EEG: Source current amplitude	EEG source imaging
Li et al., 2017 [61]	Motor	fNIRS: Motor EEG: Whole	fNIRS: HBO/HbR concentrations and slope EEG: Wavelet transform coefficients	Binary classification
Li et al., 2019 [27]	Working memory	fNIRS: Frontal, central EEG: Whole	fNIRS: HbO t-statistic EEG: Functional connectivity	EEG source imaging, Brain network analysis
Li et al., 2020 [69]	Motor	fNIRS: Frontal, parietal EEG: Whole	fNIRS: HbO t-statistic EEG: Functional connectivity	EEG source imaging, Brain network analysis

### 3.4. Parallel Analysis of EEG-fNIRS

Section 3.3 and Section 3.4 describe directional integration analyses of EEG and fNIRS. However, the majority of concurrent EEG-fNIRS studies available for review focused on parallel analysis/integration of the two complementary techniques (Figure 4). Such parallel analyses of concurrent fNIRS and EEG data usually seek to investigate the interaction between fNIRS and EEG signals through feature-based fusion analyses or correlational analyses without any directional interference from the two modalities.

#### 3.4.1. Feature Fusion Based on fNIRS–EEG Signals for Classification

Hybrid fNIRS-EEG classification-based studies account for a significant portion of feature-based fusion analyses of concurrent fNIRS-EEG data. We roughly summarize these studies into two categories based on their study aims: (1) brain–computer interface (BCI) studies and, (2) characterization of typical and atypical brain functions.

The development of a BCI system allows users to control computers or external devices based directly on the modulation of brain activity. Active investigations of the benefits of hybrid EEG-fNIRS BCIs have been conducted and validated on healthy populations in a number of BCI studies [28,29,61,70]. Specifically, by fusing the features derived from two modalities, hybrid fNIRS-EEG studies have shown enhanced classification and decoding accuracy over a single modality in various tasks, such as motor imagery and execution [61,71].

On the other hand, the complementary properties of fNIRS and EEG have led to extensive investigations of the spatiotemporal hemodynamic and electrical patterns of brain activity associated with a variety of functions, such as mental workload [72,73,74,75], affective state [76], and intellectual function [77]. Similar analysis pipelines have also been adopted to identified atypical brain patterns associated with different brain disorders, from which multimodal features can be used to differentiate patients with Alzheimer’s Disease [78] and Parkinson’s Disease [79] from healthy controls.

Despite the different aims of studies within the above two categories, most studies tend to follow similar steps when processing concurrent fNIRS and EEG data, primarily consisting of feature extraction, feature fusion, and classification. Among the reviewed literature, widely used fNIRS features are commonly derived from the concentration changes of HbO and HbR, including the mean, slope, skewness, kurtosis, peak value, variance, and median of HbO/HbR [61,80,81,82]. Typical EEG features used in concurrent fNIRS-EEG analyses largely depend on the experimental tasks. In the case of a motor task, the power spectrum density and common spatial patterns are widely used [29,83,84,85,86], mainly due to the event-related desynchronization/event-related synchronization (ERD/ERS) observed in motor-evoked electrical potential [87]. Studies involving cognitive tasks usually adopt features related to band power of signals [72,82,88,89,90,91]. Additionally, the logarithmic band power features [84], time-frequency features [61,71], and amplitude-related properties [92,93,94] are often utilized in several studies involving motor and mental tasks. Definitions and calculations of these features are summarized and shown in Table 3. In terms of classification, most existing studies adopt traditional machine learning techniques such as decision tree [93,94], linear discriminant analysis (LDA) [28,29,81,82,86,89,90,91,95,96], support vector machine (SVM) [61,70,72,85], and k-nearest neighbors (KNN) [92,93,94]. Recent studies have demonstrated increasing interest in innovative deep learning techniques such as the convolutional neural network (CNN) [97] and recurrent neural networks (RNN) [98]. We refer the readers to [99,100] for a more detailed introduction of the state-of-the-art classification techniques.

#### 3.4.2. Correlational Analysis of Concurrent fNIRS–EEG Data

The well-established phenomenon of neurovascular coupling (NVC) supports the premise that regional neural activity is accompanied by electrical activity generation and concurrent metabolic variation. Therefore, correlational analyses between concurrent fNIRS–EEG recordings have been extensively explored to investigate the spatiotemporal association between hemodynamic and electrical patterns of various brain functions. Among the eighteen articles reviewed here (Table 4), correlational analyses of concurrent fNIRS–EEG have mainly focused on correlation and coherence analyses. Pearson correlation, partial correlation, and simple linear regression are commonly used measures for assessing the relationship between the event-related potential pattern in EEG and hemodynamic changes in fNIRS [101,102,103,104,105,106,107,108,109,110]. Several studies assessed the relationship between EEG and fNIRS signal through cross-correlation analysis and canonical correlation analysis (CCA) [111,112,113,114]. Compared to the Pearson correlation method, cross-correlation can capture the delayed response of the hemodynamic compensation phenomenon after neural firing, while the CCA is a statistical method to identify a linear relationship between the two modality data sets by determining the inter-subject co-variances. Frequency and phase coupling were adopted in two studies to evaluate the interaction between electrical activation and hemodynamic response, in which spectral coherence and wavelet coherence were employed as metrics to assess the neurovascular coupling [115,116]. GLM-based analysis was also utilized to model the association of fNIRS and EEG in a recent study. Chaiarelli et al. proposed a novel general linear model-based algorithm to estimate the interaction of fNIRS and EEG signal in persons with Alzheimer’s disease [117]. In the GLM, key components of the down-sampled EEG power spectrum (theta, alpha, and beta) were used as the independent variables. The fNIRS signal was treated as the dependent variable. Then the estimated β-weight was used to assess how well the frequency-specific neuronal electric activity correlated with the corresponding hemodynamic response. Similarly, Perpetuini et al. employed an entropy based GLM method to assess neurovascular coupling alternation for an Alzheimer’s disease group relative to a healthy control group [118]. Due to the significant variation in the temporal scale of two signals, the EEG signal was first convolved with the canonical hemodynamic response and then downsampled. Compared with single EEG/fNIRS-based features, neurovascular coupling-based features achieved the highest classification accuracy for AD detection.

## 4. Integrated Analysis of Concurrent fNIRS-EEG: Current Limitations and Future Directions

Both fNIRS and EEG are portable, non-invasive and cost-effective brain imaging techniques that enable researchers to study brain function in conditions not suited for other neuroimaging modalities such as fMRI and MEG. Accordingly, acquisition and analysis of concurrent, integrated fNIRS–EEG data can potentially reveal more comprehensive information associated with brain activity. The present review highlights what data processing and analysis approaches can be adopted to study brain functioning in healthy cohorts as well as those with brain disorders, thus serving as a foundation for future work. However, it should be acknowledged that further development of integrated analyses of the two modalities is required to fully benefit from the added value of each modality.

Neurovascular coupling in the brain is highly dynamic in nature, for both resting state and task-engaging states. While various fusion approaches of fNIRS and EEG signals allow for the imaging and investigation of brain activity with richer information, the majority of such integrated analyses still rely on a summary of signals extracted from fNIRS and EEG time series data. Neural activity is time-varying, thus requiring a more dynamic analytic approach to improve accuracy in modeling actual brain function. Therefore, it is important to explore the dynamic interaction of fNIRS and EEG signals with a more fine-grained temporal resolution. This is a challenge for fNIRS signals, which usually suffer reduced temporal resolution relative to EEG. Recently, effort has been made to tackle this challenge by growing interest in the temporal fluctuations of fNIRS-based functional connectivity across the brain, the so-called dynamic functional connectivity (dFC). Several studies have shown that resting-state and task-evoked hemodynamic responses can be characterized using dFC analysis to reflect a more dynamic and modular nature of neurovascular coupling during normal cognitive processing and atypical brain activity associated with Alzheimer’s disease [119,120]. It is expected that fusion of the dynamic properties of fNIRS and EEG may open new lines of concurrent fNIRS–EEG analyses.

Despite the numerous approaches for integrated analysis of concurrent fNIRS–EEG, most studies have utilized feature-based fusion of these two modalities, such as hybrid BCI systems or correlation analyses between fNIRS-based (e.g., mean HbO) and EEG-based features (e.g., power spectrum). Such analyses only allow for a rough characterization of neurovascular coupling underlying brain activity. Questions remain as to how the findings obtained from the integrated analyses of fNIRS–EEG reflects the interaction between neuronal electric activity and the resulting hemodynamic response. Therefore, it is expected that more directionally integrated analyses of fNIRS and EEG data, such as the fNIRS-informed EEG analyses or the EEG-informed fNIRS analyses, can be explored in future work.

Combining fNIRS and EEG serves to bridge brain imaging techniques across laboratory settings to practical applications due to their high mobility, non-invasiveness, and low cost compared to MRI-based techniques. However, few studies, especially those focusing on hybrid fNIRS–EEG BCI systems, have validated the feasibility of using such multimodal approaches to address the needs of multiple practical scenarios, such as hybrid real-time BCI systems, bedside monitoring, or neuromodulation based on the so-called brain controllability analysis, to treat different neurological and psychiatric diseases [121,122,123]. Therefore, a prioritized goal of future research may focus on enhancing the ecological validity of experimental designs and analysis pipelines/algorithms that can be adopted in online or low time-delayed settings. In fact, as motivated by real-time BCI applications, progress has been made to increase the temporal response of fNIRS–BCI systems through single-trial analysis [124], early signal detection [61], and adaptive filtering [125]. We anticipate future solutions for real-time fNIRS signal processing may facilitate the development of real-time hybrid BCI systems that enable human–computer interaction with high spatial and temporal performance. Another typical experimental protocol of fNIRS is hyperscanning, where brain activities are recorded from two or more participants simultaneously, permitting a direct investigation of how multi-brains communicate to each other during social interaction [120,126]. Following this, we expect that the development of wearable fNIRS and EEG devices will likely drive the typical fNIRS-based hyperscanning studies toward multimodal fNIRS–EEG system-based hyperscanning research. This innovation will enable us to examine human interaction in a high spatiotemporal resolution perspective, thereby expanding our understanding of the neural mechanism underlying social interaction.

Apart from the perspective on methodological integration of fNIRS and EEG, we want to highlight challenges in instrument development that might affect study design and signal processing of concurrent fNIRS–EEG studies. In particular, conventional concurrent fNIRS–EEG studies usually connect separate fNIRS and EEG systems for data recording, which reduces the mobility of both systems and constrains the applications of concurrent fNIRS and EEG. Recent advances have been made toward fiberless and wearable integrated fNIRS–EEG systems that allows for broader research scenarios such as social interaction and outdoor activity [127,128]. However, further improvement of fNIRS and EEG instruments is necessary when applying these systems in clinical cohorts with psychological or psychiatric disorders. For example, patients with psychiatric disorders, such as ASD and ADHD, often display motor restlessness, anxiety, or hyperarousal symptoms that require specific considerations during development of integrated fNIRS–EEG instrumentation. Key factors to be considered may include (1) user-friendly materials for comfort contact between electrodes/optodes, (2) lightweight/highly integrated design for enhanced measurement experience, and (3) advanced signal processing algorithms for robust long-time real-world study. In addition, simultaneous multimodal data recording, including brain, physiological, and behavioral information, is important to the comprehensive understanding of disease-linked/function-specific brain activity. Physiological or auxiliary signals (e.g., blood pressure, respiration, and head movement) have been proven to greatly improve the filtering of physiological interference and motion artifacts during fNIRS signal processing [129,130,131]. In this context, one impactful direction of fNIRS–EEG instrument development should focus on the development of multimodal systems that are deeply integrated with these and other emerging modalities, such as eye tracking devices, physiology modules (e.g., heart rate, skin conductivity), and accelerometers as well as VR devices. From a clinical perspective, such multimodal systems could offer multi-dimensional brain–physiology–behavior biomarkers specifically linked to brain disorders at individual level. Together with powerful statistical/machine learning, we expect that future studies in the field will propose advanced algorithms to fuse such multimodal information for accurate monitoring of brain activity and facilitating personalized treatment protocols to obtain enhanced efficiency for each individual patient.

## Figures and Tables

**Table 1:** Characteristics of studies that performed EEG-informed fNIRS analysis.

Authors	Tasks	Brain Regions	Features	Analysis Methods
Peng et al., 2014 [51]	Resting	fNIRS: Whole EEG: Whole	fNIRS: HbO/HbR/HbT concentration EEG: Amplitude	GLM
Pouliot et al., 2014 [52]	Resting	fNIRS: Whole EEG: Whole	fNIRS: HbO/HbR/HbT concentration EEG: Amplitude	GLM
Talukdar et al., 2015 [53]	Resting	fNIRS: Whole EEG: Whole	fNIRS: HbO concentration EEG: Power spectral envelopes	GLM
Peng et al., 2016 [54]	Simulation; Resting	fNIRS: Whole EEG: Whole	fNIRS: HbO/HbR/HbT concentration EEG: Amplitude	GLM
Khan et al., 2018 [55]	Motor	fNIRS: Left motor EEG: Left motor	fNIRS: HbO/HbR concentration EEG: Power spectrum	Vector-phase analysis
Zama et al., 2019 [56]	Motor	fNIRS: Motor EEG: Whole	fNIRS: HbO/HbR concentration EEG: ERD/ERS	GLM
Li et al., 2020 [57]	Motor	fNIRS: Motor EEG: Whole	fNIRS: HbO/HbR concentration EEG: Absolute Power (amplitude)	GLM
Sirpal et al., 2021 [58]	Resting	fNIRS: Whole EEG: Whole	fNIRS: HbO concentration EEG: Amplitude	Autoencoder

**Table 3:** Definition and calculation of EEG and fNIRS features.

Features	Definitions
Mean (*µ*)	$µ=\frac{1}{N}\overset{t_{2}}{\underset{t=t_{1}}{\sum }}x\left(t\right)$
Slope (*Sp*)	$Sp=\frac{x\left(t_{2}\right)-x\left(t_{1}\right)}{t_{2}-t_{1}}$
Standard deviation (*Sd*)	$Sd=\sqrt{\frac{\sum {\left(x\left(t\right)-µ\right)}^{2}}{N}}$
Skewness (*Skew*)	$Skew=\frac{1}{N}\frac{\sum_{t=t_{1}}^{t_{2}}{\left(x\left(t\right)-µ\right)}^{3}}{Sd^{3}}$
Kurtosis (*Kurt*)	$Kurt=\frac{1}{N}\frac{\sum_{t=t_{1}}^{t_{2}}{\left(x\left(t\right)-µ\right)}^{4}}{Sd^{4}}$
Median (*Med*)	$Med=\{\begin{array}{c} x\left(\frac{n}{2}\right)\;\;\;\;\;\;\;\;\;\;\;\;\mathrm{if}\;n\;\mathrm{is}\;\mathrm{even}\\ \frac{x\left(\frac{n-1}{2}\right)+x\left(\frac{n+1}{2}\right)}{2}\;\;\mathrm{if}\;n\;\mathrm{is}\;\mathrm{odd} \end{array}$
Power spectral density (*PSD*)	$PSD_{f}^{t}=\frac{1}{N}\overset{N}{\underset{t=1}{\sum }}{\left|\;x\left(t\right)e^{-2\pi ft}\right|}^{2}$
Logarithmic band power (*PLB*)	$PLB_{f}=log\left(PSD_{f}\right)$
Common spatial pattern (*CSP*)	$X_{i}=\left[C_{i}C_{M}\right]\left[\begin{array}{c} S_{i}\\ S_{M} \end{array}\right],\;\left(i=1,2\right)$
Phase locking value (*PLV*)	$PLV=\left|N^{-1}\overset{N}{\underset{t=1}{\sum }}e^{i(\emptyset x\left(t\right)-\emptyset y\left(t\right)}\right|$
Pearson correlation coefficient (*r*)	$r=\frac{\sum \left(x\left(t\right)-\bar{x}\right)\;\left(y\left(t\right)-\bar{y}\right)}{\sqrt{\sum {\left(x\left(t\right)-\bar{x}\right)}^{2}\sum {\left(y\left(t\right)-\bar{y}\right)}^{2}\;\;}}$

*x*(*t*) is the input brain signals (i.e., EEG and fNIRS). N is the number of observations of the samples. ∅x(t) and ∅y(t) are instantaneous phase values at time point *t*. *f* refers to the *f*-th frequency band. *X_i_* represent the measured signals of *i*-th tasks. *S_i_* is the source signal related to the *i*-th task. *S_M_* is the common source signal of both signals. *C_i_* and *C_M_* are the weight matrix of common spatial pattern. *x*(*t*) and *y*(*t*) present the signals from different channel. $\bar{x}$ and $\bar{y}$ refer to the mean value of the signals of *x*(*t*) and *y*(*t*)*,* respectively.

**Table 4:** Studies using parallel EEG–fNIRS analysis for neurovascular coupling investigation.

Authors	Task	Brain Regions	Features	Correlation Method
Chen et al., 2015 [101]	Visual and auditory	fNIRS: Temporal, occipital EEG: Whole	fNIRS: HbO/HbR concentrations EEG: ERP	Pearson correlation
Chen et al., 2020 [102]	Resting	Whole	fNIRS: HbO/HbR global amplitude EEG: Power Spectrum	Partial correlation
Balconi et al., 2016 [103]	Visual and auditory	fNIRS: Frontal EEG: Whole	fNIRS: HbO concentrations EEG: ERP	Pearson correlation
Zich et al., 2017 [104]	Motor execution	Central	fNIRS: HbO/HbR concentrations EEG: ERD	Pearson correlation
Borgheai et al., 2019 [105]	Mental arithmetic	fNIRS: Frontal EEG: Whole	fNIRS: HbO/HbR concentrations EEG: Power spectrum and ERP	Pearson correlation
Gentile et al., 2020 [106]	Finger tapping	fNIRS: Motor EEG: Whole	fNIRS: HbO/HbR concentrations EEG: ERP	Linear regression
Zhang et al., 2020 [107]	Resting	Whole	fNIRS: dynamic functional connectivity EEG: Microstate (amplitude)	Pearson correlation
Lin et al., 2020 [108]	Mental	Occipital and parietal	fNIRS: HbO concentration EEG: Power spectrum and ERD	Pearson correlation
Kaga et al., 2020 [109]	Working memory	fNIRS: Frontal EEG: Pz, Cz, Pz,	fNIRS: HbO concentration EEG: ERP	Pearson correlation
Suzuki et al., 2018 [110]	Working memory	fNIRS: Frontal EEG: Fz, O1, O2,	fNIRS: HbO concentration EEG: Power spectrum	Pearson correlation
Keles et al., 2016 [111]	Resting	Whole	fNIRS: HbO/HbR concentrations EEG: Power spectrum	Cross-correlation
Pinti et al., 2021 [112]	Visual stimulation	Occipital	fNIRS: HbO/HbR concentrations EEG: Power spectrum	Cross-correlation
Nair et al., 2021 [113]	Anesthesia	Frontal	fNIRS: HbO/HbR amplitude EEG: Amplitude	Cross-correlation and phase difference
Al-Shargie et al., 2017 [114]	Mental arithmetic	Frontal	fNIRS: HbO concentration EEG: Average power (amplitude)	Canonical correlation analysis
Govindan et al., 2016 [115]	Resting	Frontotemporal	fNIRS: difference between HbO and HbR EEG: Amplitude	Coherence and Phase Spectra
Chalak et al., 2017 [116]	Resting	Parietal	fNIRS: Cerebral tissue oxygen saturation EEG: Amplitude	Wavelet coherence
Chiarelli et al., 2021 [117]	Resting	Whole	fNIRS: HbO/HbR concentrations EEG: Power envelops	GLM-Standardized β-weight
Prepetuini et al., 2020 [118]	Working memory	fNIRS: Frontal EEG: Whole	fNIRS: HbO/HbR sample entropy EEG: Sample entropy	GLM-Standardized β-weight

## Data Availability

Not applicable.
